# Supplementary material for: Revision of the Afro-Madagascan genus Costularia (Schoeneae, Cyperaceae): infrageneric relationships and species delimitation
Source: PeerJ. 2019 Feb 27;7:e6528. doi: 10.7717/peerj.6528 (PMC6397637; doi:10.7717/peerj.6528)
Supplement: Supplemental Information 1 — *indicates new accessions; a dash (–) indicates missing data. [file peerj-07-6528-s001.docx]

| **Species** | **Voucher** | **Distribution** | **ETS** | **ITS** | ***trnL-F*** |
| --- | --- | --- | --- | --- | --- |
| **Ingroup** |  |  |  |  |  |
| *Costularia andringitrensis* Larridon | I. Larridon et al. 2010-0219 (GENT) | C. Madagascar | MH512812***** | MH512829***** | MH512841***** |
| *Costularia cadetii* Larridon | M. Luceño 4ML09 (UPOS) | La Réunion | MH512813***** | MH512830***** | MH512842***** |
| *Costularia humbertii* Bosser | H. Humbert 23767 (P01868312) | Madagascar | MF315017 | – | – |
| *Costularia itremoensis* Larridon | H. Humbert & C.F. Swingle 4995 (P) |  | MH512814***** | MH512831***** | MH512843***** |
|  | H. Humbert 30060 (P) |  | MH512815***** | MH512832***** | – |
| *Costularia leucocarpa* (Ridl.) H.Pfeiff. | I. Larridon et al. 2010-0237 (GENT) | Madagascar | MF315025 | MF314960 | MF314977 |
|  | C. Rakotovao 3556 (GENT) |  | MH512817***** | – | MH512845***** |
| *Costularia melicoides* (Poir.) C.B.Clarke | H.J. Lam & Meeuse 5257 (L) | Mascarenes | MF315020 | MF314961 | MF314978 |
|  | M. Luceño 128ML08 (UPOS) |  | MH512818***** | MH512833***** | MH512846***** |
|  | M. Luceño 17ML09 (UPOS) |  | MH512819***** | MH512834***** | MH512847***** |
| *Costularia melleri* (Baker) C.B.Clarke ex Cherm. | I. Larridon et al. 2010-0249 (GENT) | C. Madagascar | MF315024 | MF314962 | MF314979 |
| *Costularia natalensis* C.B.Clarke | J. Browning 531 (GENT) | Malawi to S. Africa | MF315026 | MF314963 | MF314980 |
|  | G.A. Verboom 773 (BOL) |  | KF553542 | KF553445 | – |
| *Costularia pantopoda* (Baker) C.B.Clarke ex Cherm. var. *pantopoda* | I. Larridon et al. 2010-0144 (GENT) | C. Madagascar | MF315021 | MF314965 | MF314982 |
| *Costularia pantopoda* var. *baronii* (C.B.Clarke) Kük. | I. Larridon et al. 2010-0153 (GENT) | WC. & C. Madagascar | MF315022 | MF314966 | MF314983 |
|  | I. Larridon et al. 2010-0139 (GENT) |  | MH512822***** | MH512836***** |  |
| *Costularia pantopoda* var. *robusta* (Cherm.) Kük. | L.J. Razafitsalama 431 (P) | C. Madagascar | MF315023 | MF314967 | MF314984 |
|  | M.W. Callmander et al. 445 (P) |  | MH512823***** | – | – |
| *Costularia cf. pantopoda* | H. Humbert 30061 (P) | C. Madagascar | MH512820***** | MH512835***** | MH512848 |
| *Costularia purpurea* Cherm. | W. Armand 74 (P01898127) | C. Madagascar | MF315019 | MF314968 | MF314986 |
|  | L. Nusbaumer & P. Ranirison 1677 (G) |  | MH512824***** | MH512837***** | MH512848***** |
|  | L. Nusbaumer & P. Ranirison 1754 (G) |  | MH512825***** | MH512838***** | MH512850***** |
| (*Costularia laxa* Cherm.) | C. Rakotovao 3033 (BR) | Madagascar | MF315018 | MF314959 | MF314976 |
|  | I. Larridon et al. 2010-0219 (GENT) |  | MH512816***** | MH512828***** | MH512844***** |
| *Costularia xipholepis* (Baker) Henriette & Senterre | B. Senterre & E. Henriette 6964 (GENT) | Seychelles (Mahé) | MF315027 | MF314969 | MF314969 |
|  | B. Senterre & E. Henriette 7101 (GENT) |  | MH512826***** | MH512839***** | MH512851***** |
|  | M. Luceño 8406ML (UPOS) |  | MH512827***** | MH512840***** | – |
| **Outgroup** |  |  |  |  |  |
| *Capeobolus brevicaulis* (C.B.Clarke) Browning | G.A. Verboom 646 (BOL) | Cape Prov. | KF553535 | KF553443 | DQ058303 |
| *Chamaedendron fragilis* (Däniker) Larridon | G.L. Webster & R. Hildreth 14967 (P06866925) | SE. New Caledonia | MF315014 | MF314958 | MF314974 |
| *Chamaedendron* *kuekenthaliana* Larridon | H.S. McKee 31395 (P06866148) | EC. New Caledonia | MF315013 | – | – |
| *Chamaedendron nervosa* (J.Raynal) Larridon | T. Jaffré 2378 (P02204080) | New Caledonia | MF315015 | MF314964 | MF314981 |
| *Chamaedendron xyridioides* (Däniker) Larridon | J.J. Bruhl 3260 (NE) | SE. New Caledonia | MF315016 | MF314970 | MF314991 |
| *Cyathocoma hexandra* (Nees) Browning | G.A. Verboom 648 (BOL) | SW. & S. Cape Prov. | KF553550 | – | DQ058304 |
| *Oreobolus acutifolius* S.T.Blake | Okenden s.n. (K) | Tasmania | MF315002 | DQ450466 | DQ456956 |
| *Oreobolus obtusangulus* Gaudich. | Moore 2817 (K) | Peru to Falkland Is. | – | DQ450472 | DQ456962 |
| *Oreobolus pumilio* R.Br. | J.J. Bruhl 1879C (NE) | New Guinea, SE. Australia | MF315006 | DQ450476 | DQ456966 |
