# Supplementary material for: Revision of the Afro-Madagascan genus Costularia (Schoeneae, Cyperaceae): infrageneric relationships and species delimitation
Source: PeerJ. 2019 Feb 27;7:e6528. doi: 10.7717/peerj.6528 (PMC6397637; doi:10.7717/peerj.6528)
Supplement: Supplemental Information 3 [file peerj-07-6528-s003.docx]

**1. *Costularia andringitrensis*** Larridon

Madagascar, **Fianarantsoa:** Haute Matsiatra, Andringitra Mts, 2000–2500 m, 27 November 1924 – 08 December 1924, *H. Humbert 3784* (K!, MNHN-P-P01898149!, MNHN-P-P01898161!).

**2. *Costularia baronii*** C.B.Clarke

Madagascar, **Antananarivo:** Vakinankaratra, Antsirabe, 2200 m, May 1962, *H. Perrier de la Bâthie 2566* (MNHN-P-P01898167!); Ibity, 13 October 1970, *M. Keraudren 24566* (MNHN-P-P01898143!); Ankaratra, 1800 m, February 1964, *J.M. Bosser 18845* (MNHN-P-P01898160!, TAN). **Fianarantsoa:** Ivakoany, 1300–1640 m, 17–18 December 1928, *H. Humbert 7008* (MNHN-P-P01898151!, MNHN-P-P01898152!); Pic d'Ivohibe, 1500–2000 m, 5–11 November 1924, *H. Humbert 3344* (MNHN-P-P01868278!); Haute Matsiatra, Andringitra National Park, 22°07'28.0"S, 46°52'32.7"E, 2063 m, 18 April 2010, *I. Larridon, W. Huygh, M. Reynders, A.M. Muasya & V. Randrianasolo 2010-0139* (GENT!, TAN!); Haute Matsiatra, Andringitra National Park, 22°08'14.1"S, 46°52'00"E, 2161 m, 18 April 2010, *I. Larridon, W. Huygh, M. Reynders, A.M. Muasya & V. Randrianasolo 2010-0153* (GENT!, TAN!). **Toliara:** Fort-Dauphin, Andohahela, 24°33'42"S, 46°43'18"E, 1875 m, 4 December 1995, *P.J. Rakotomalaza 563* (EIU, K!, MO, P, TAN); Andohahela Mts, 1700–1975 m, January 1934, *H. Humbert 13645* (MNHN-P-P01898145!); Amboasary Atsimo, Andohahela, 24 December 1959, *Réserves Naturelles 10403* (MNHN-P-P01898154!, MNHN-P-P01898155!).

**3. *Costularia brevifolia*** Cherm.

Madagascar, **Fianarantsoa:** Midongy du Sud AP, 25 August 1926, *R. Decary 4999* (MNHN-P-P01868313!, MNHN-P-P01868314!). **Toliara:** Mananara Bassin, May 1920, *H. Perrier de la Bâthie 12643bis* (MNHN-P-P00459975!, MNHN-P-P00459976!, MNHN-P-P00459977!); Anosy, Beampingaratra, 600–800 m, 31 October 1928–1 November 1928, *H. Humbert 6290* (MNHN-P-P01868316!, MNHN-P-P01868317!, MNHN-P-P01868318!); Anosy, Mandrare, 900 m, January–February 1934, *H. Humbert 13892* (MNHN-P-P01868263!, MNHN-P-P01868319!, MNHN-P-P01868320!, MNHN-P-P01868321!); Anosy, Mandrare, 200–850 m, 11–16 March 1947, *H. Humbert 20470* (MO, MNHN-P-P01868315!, MNHN-P-P02640227!); Anosy, Fort Dauphin, Forêt de Tsitongambarika, 24°35'44"S, 47°02'43"E, 846 m, 23 November 2009, *R. Razakamalala 4866* (K!, MO, TAN!).

**4. *Costularia cadetii*** Larridon

La Réunion, **Saint-Benoît:** Plaine des Sables, 2200 m, 14 February 1969, *T. Cadet 1973* (MAU0003581!, REU015245!); Plateau des Basaltes (Plaine des Remparts), 2400 m, 31 May 1965, *T. Cadet 454* (MAU0003579!, MAU0003580!, REU015249!); December 2004, *J. Dupont 2397* (MNHN-P-P06869805!); Piton de la Fournaise, 21°13.3333 S, 55°41.3551 E, 2300–2350 m, 1 January 2009, *M. Luceño & M. Guzmán 4ML09* (UPOS!). **Sainte Clotilde:** Plaine de Chicots, Roche Ecrite, [close to the Gîte], 1700–2277 m, 7 January 1975, *L. Bernardi 14892* (G, K!, MNHN-P-P03471923!). **Saint-Paul:** Grand Bénare, environs du Maïdo, 2100 m, 22 November 1968, *T. Cadet 1764* (REU015246!).

**5. *Costularia humbertii*** Bosser

Madagascar, **Antsiranana:** Marojejy, 1850–2137 m, 17–20 December 1948, *H. Humbert 22677* (MNHN-P-P01868309!); Marojejy, 1400–1450 m, 17–22 March 1949, *H. Humbert 23610* (MNHN-P-P01868311!); Marojejy, 1800–2137 m, 26 March – 2 April 1949, *H. Humbert & G. Cours* 23767 (MNHN-P-P01868312!); Marojejy, 2000 m, 27 March 1949, *G. Cours 3484* (MNHN-P-P01868310!, TAN); Marojejy, 2137 m, November 1973, *P. Morat 4063* (MNHN-P-P01868308!); Marojejy NR, 1900–2133 m, 15 February 1989, *J.S. Miller & P. Lowry 4175* (GENT!, MO, TAN).

**6. *Costularia itremoensis*** Larridon

Madagascar, **Fianarantsoa:** Amoron'i Mania Region, Mountains W of Itremo (W Betsileo), 1500–1700 m, 17–22 January 1955 & 18–22 April 1955, *H. Humbert 30060* (MNHN-P-P01875040!); Amoron'i Mania Region, Itremo Massif, on Route Nationale 35, 46 km W of Ambatofinandrahana, 1650 m, 14 March 1992, *P.B. Phillipson et al. 3886* (K!, MO); Massif de l'Itremo, vicinity of col de Itremo, 1500–1685 m, 27 January 1975, *T.B. Croat 29800* (MNHN-P-P01898132!); P.K. 10 entre Ivato et Ambatofinandrahana, September 1956, *J.M. Bosser 9802* (MNHN-P-P01898134!); Isalo North, 1300 m, 4 June 1931, *E. Basse s.n.* (K!).

**7. *Costularia leucocarpa*** (Ridl.) H.Pfeiff.

Madagascar, **Antananarivo:** Ankeramadinika, January, *G.F. Scott Elliot 1777* (K!, MNHN-P-P06869800!); March 1922, *Waterlot 460* (MNHN-P-P06869799!); Mantasoa (Imerina), *Le Myre de Vilers s.n.* (MNHN-P-P06869803!); Analamanga, Ambatolaona, 21 January 1917, *R. Decary s.n.* (MNHN-P-P06869797!). **Antsiranana:** Manongarivo, 800 m, 1909, *H. Perrier de la Bâthie 2623* (MNHN-P-P06869793!); Tsaratanana, 2000 m, *H. Perrier de la Bâthie 16393* (MNHN-P-P06869794!); Tsaratanana, 2000 m, January 1923, *H. Perrier de la Bâthie 15655* (MNHN-P-P06869795!); SAVA, Sambava, Bevontro, Tsaratanana, Morafeno, 14°10’33”S, 49°18’38”E, 907 m, 7 February 2007, *C. Rakotovao et al. 3556* (G, GENT!, MO, MNHN-P-P06869817!, TAN); SAVA, Andapa, 14°10'47"S, 49°22'01"E, 1567 m, 20 February 2007, *D. Ravelonarivo et al. 2181* (G, K!, MO, P, TAN!). **Fianarantsoa:** Amoron'i Mania, Ambohimitombe forest, 1350–1440 m, 31 December 1894, *C.I. Forsyth Major 224* (K!); Amoron'i Mania, Ambohimitombe forest, 1350–1440 m, 22 December 1894, *C.I. Forsyth Major 254* (G, K!); Amoron'i Mania, Ambohimitombe forest, 1350–1440 m, 1 January 1895, *C.I. Forsyth Major 257* (G, K!); Vatovavy-Fitovinany, Ranomafana NP, Amboditanimena Circuit, 21°12’12.5”S, 47°22’10.0”E, 1205 m, 21 April 2010, *I. Larridon, W. Huygh, M. Reynders, A.M. Muasya & V. Randrianasolo 2010-0237* (GENT!, TAN!); Vatovavy-Fitovinany, Ranomafana NP, Ankevohevo, 19 December 1965, *J. & M. Peltier 5527* (MNHN-P-P06869819!); 34 km N of Fianarantsoa, PK 376 on Route #7, 31 January 1975, *T.B. Croat 30132a* (MO, MNHN-P-P06869812!); Itomampy basin (south east), Mt Papanga, near Befotaka, 1300–1700 m, 2–3 December 1928, *H. Humbert 6900bis* (MNHN-P-P01866845!, MNHN-P-P06869796!); Ankafina-Tsarafidy, 1250–1260 m, 31 January 1975, *T.B. Croat 30132* (TAN!). **Mahajanga:** Mangindrano, Ambohimirahavavy, 2000–2200m, 19 January 1951 – 12 February 1951, *H. Humbert 25211* (MNHN-P-P01866863!, MNHN-P-P06869809!). **Toamasina:** Moramanga, Ambohibary, Ampitambe, Ambatovy, 18°51’16”S, 48°18’26”E, 1108 m, 19 February 2005, *C. Rakotovao et al. 1205* (K!, MO, P, TAN); Moramanga, Andasibe, Menalamba, Ambatovy forest, 18°51’06”S, 48°19’06”E, 1114 m, 12 January 2005, *P. Antilahimena 3104* (MO, MNHN-P-P06869813!, TAN); Moramanga, Andasibe, Menalamba, Analamay, 18°48’29”S, 48°20’20”E, 1080 m, 21 December 2005, *P. Antilahimena et al. 4511* (MO, MNHN-P-P06869816!, TAN); Moramanga, Ampitambe, Ambatovy, Ambohibary, 18°47’59”S, 48°20’01”E, 1070 m, 19 December 2004, *R. Ranaivojaona et al. 999* (K!, MO, MNHN-P-P06869814!, TAN); Moramanga, Ampitambe, Ambatovy, Ambohibary, 18°51’16”S, 48°18’46”E, 1070 m, 22 February 2005, *H. Razanatsoa et al. 255* (MO, MNHN-P-P06869815!, TAN); E of Moramanga, Andasibe, Perinet Reserve, 5 March 1988, *D.A. Simpson 88/111* (K!, TAN); Moramanga, Andasibe, Perinet (East), February 1955, *J. Bosser 7773* (K!, MNHN-P-P06869802!); S of Moramanga, 6 March 1942, *R. Decary 17773* (MNHN-P-P06869811!); Alaotra-Mangoro, Lake Alaotra, ANK 35 (MNHN-P-P06869818!, TAN); ANK 10 (MNHN-P-P06869801!); Alaotra-Mangoro, Lake Alaotra, ANK 103 (MNHN-P-P06869798!, TAN); Alaotra-Mangoro, Anosibe, 17 February 1930, *R. Decary 7156* (MNHN-P-P06869792!). **Toliara:** Mandrare valley, tributary of the Manampanihy (south east), Mt S of Tanandava, 11–16 March 1947, *H. Humbert 20471* (MNHN-P-P06869810!).

**8. *Costularia melicoides*** (Poir.) C.B.Clarke

La Réunion, **Saint-Denis:** Le Brûlé, April 1956, *J. Bosser 9344* (MNHN-P-P01866834!, MNHN-P-P01866835!, MNHN-P-P00552876!); Le Brûlé, 1300–1500 m, 22 October 1972, *J. Bosser 21329* (K!, MNHN-P-P01866842!, MNHN-P-P03471928!). **Saint-Benoît:** Salazie, Sentier de Grand Ilet à la Roche Ecrite, 1700 m, 15 October 1974, *T. Cadet 4831* (MNHN-P-P00552882!, REU015241!); Salazie, Bélouve, 1500 m, June 1957, *J. Bosser 11682* (MNHN-P-P01866837!, MNHN-P-P00552877!); Bélouve, 2000 m, June 1957, *J. Bosser 11929* (MNHN-P-P00552874!); Bélouve, 1 November 1938, *H.J. Lam & Meeuse* 5257 (L1368036!), Bébour, 27 May 1976, *J. Bosser 22230* (MNHN-P-P01866841!, MNHN-P-P00552875!); Bébour, 1300 m, 7 November 1967, *T. Cadet 1143* (MNHN-P-P00552878!, REU015248!); Cirque de Takamaka, 900 m, 4 January 1972, *T. Cadet 3418* (MNHN-P-P06869804!, REU015243!); Takamaka, 800–900 m, 2 November 1972, *J. Bosser 21472* (MNHN-P-P00552891!); Plaine des Sables, 2000 m, 27 July 1962, *T. Cadet 416* (MAU0003576!, REU015251!). **Saint-Paul:** Trois-Bassins, Grand Bénare, 01 February 1847, *L.H. Boivin s.n.* (MNHN-P-P01866838!, MNHN-P-P01866856!, MNHN-P-P01866857!); Trois-Bassins, Grand Bénare, 15 February 1847, *L.H. Boivin 998* (MNHN-P-P03471925!; MNHN-P-P01866839!, MNHN-P-P01866840!, MNHN-P-P00552884!, MNHN-P-P06869806!). **Saint-Pierre:** Saint-Philippe, Mare Longue, 500–600 m, 15 December 1971, *J. Bosser 20842* (K!, MNHN-P-P01866843!, MNHN-P-P00552892!); Saint-Philippe, Basse-Vallée, 900 m, 22 November 1973, *T. Cadet 4525* (MNHN-P-P00552883!); Le Tévelave, 1200 m, October 1965, *T. Cadet 441* (MAU0003577!, MNHN-P-P01866860!, MNHN-P-P01866861!, MNHN-P-P03471929!, REU015250!); Nez Coupé du Tremblet (volcan), 1900 m, 16 April 1971, *T. Cadet 3220* (REU015244!); Sentier du Tremblet, 9 November 1972, *J. Bosser 21515* (MNHN-P-P00552890!); La Plaine des Cafres, Col de Bellevue, 1605–1615 m, 21°9.9754 S, 55°35.3453 E, 31 December 2008, *M. Luceño* *& M. Guzman 128ML08* (UPOS!); Les Makes, La Fenêtre, 1500–1650 m, 21°11.1304 S, 55°25.34634 E, 5 January 2009, *M. Luceño* & *M. Guzman* *17ML09* (UPOS!). **Without locality/district:** Sentier … à la Roche Ecrite, 11 November 1977, *F. Friedmann 3274* (MNHN-P-P03471924!); *L.H. Boivin 255* (MNHN-P-P00552885!); *L.H. Boivin s.n.* (MNHN-P-P03471917!, MNHN-P-P01866858!; MNHN-P-P06869807!, MNHN-P-P06869808!); *A. Richard 528* (MNHN-P-P00552886!); *Commerson s.n.* (MNHN-P-P03471926!); Plateau…, 19 July 1875, *M.G. de l’Isle 352* (MNHN-P-P00552888!, MNHN-P-P01866859!); Grande Montée, September 1866, *Unknown collector s.n.* (MNHN-P-P00552917!); *Unknown collector s.n.* (MNHN-P-P00552887!); *Unknown collector s.n.* (MNHN-P-P03471930!, MNHN-P-P00552889!).

Mauritius, **Flacq:** Le Grand Fond, 280 m, 17 June 1890, *H.H. Johnston s.n.* (K000244879!, MAU0003574!). **Plaine Wilhems:** Petrin, 27 January 1998, *M. Virahsawmy s.n.* (MAU0003571!, MAU0003572!); “Perrier” [Petrin], 8 October 1941, *R.E. Vaughan s.n.* (MAU0003575!); 20°23.51’S, 57°27.70’E, 600 m, 7 December 1998, *D. Florens s.n.* (MAU0003573!).

**9. *Costularia melleri*** (Baker) C.B.Clarke ex Cherm.

Madagascar, **Antananarivo:** Analamanga, Manjakandriana, S of Ambatolaona, 1400 m, 10 December 1912, *R. Viguier & H. Humbert 1974* (MNHN-P-P01868293!, MNHN-P-P01868294!, MNHN-P-P01868295!); Ankazobe, September 1955, *J.M. Bosser 8342* (TAN!). **Fianarantsoa:** near Fianarantsoa, 1200 m, 27 October 1926, *R. Decary 5826* (MNHN-P-P01868296!); Vatovavy-Fitovinany, Ranomafana NP, Amboditanimeba Circuit, 21°11'01.4"S, 47°23'21.9"E, 1178 m, 21 April 2010, *Larridon et al. 2010-0249* (GENT!, TAN). **Toamasina:** Soanierana Ivongo, November 1954, *J. Bosser 122* (MNHN-P-P01868297!); Ambila-Lemaitso, 7 March 1988, *D.A. Simpson 88/129* (K!); Maningory, 14 December 1944, *A.-M. Homolle 1853Bis* (P!). **Locality unknown:** January 1882, *R. Baron 1026* (K!); *R. Baron 2846* (K!, K!, MNHN-P-P01868289!); November 1885, *R. Baron 4104* (K!); *Richard s.n.* (MNHN-P-P01868290!); *L.M.A. Du Petit Thouars s.n.* (MNHN-P-P01868291!, MNHN-P-P01868292!).

**11. *Costularia natalensis*** C.B.Clarke

Malawi, **Southern Region:** Mulanje District, Mt Mulanje, Litchenya Plateau, 1820 m, 05 July 1946, *L.J. Brass 16673* (K!, NY); Mulanje District, Mt Mulanje, Litchenya Plateau, 1890 m, 08 July 1946, *L.J. Brass 16740* (K!, NY); Mulanje District, Mt Mulanje, 11 February 1958, *J.B. Chapman 479* (K!); Mulanje District, Mt Mulanje, Litchenya Plateau, 2150 m, 11 June 1962, *E.A. Robinson 5331* (K!); Mulanje District, Mt Mulanje, 2130 m, 06 April 1970, *R.K. Brummitt 9668* (K!); Mulanje District, Mt Mulanje, Chambe Plateau, 1860 m, 12 February 1979, *S. Blackmore et al. 393* (K!); Mulanje District, Mt Mulanje, Litchenya Plateau, 1830 m, 08 February 1981, *J.D. Chapman & E.J. Tawakali 5529* (K!, MAL); Mulanje District, Mt Mulanje, 2000 m, 02 March 1987, *J.D. & E.G. Chapman 8367* (K!, MO); Mulanje District, Mt Mulanje, 1874 m, 28 May 2005, *H.T. Chapama 253* (K!).

Mozambique, **Manica Province:** Manica District, Chimanimani Mts, 2000 m, 31 May 1969, *T. Muller 1257* (K!, SRGH); Manica District, Chimanimani Mts, Northern slopes between Mt Dombe & Mt Peza, 19.743530°S, 33.014972°E, 1582 m, 17 April 2014, *J. Timberlake 5966* (BR!, K!, LMA, SRGH); Manica District, Chimanimani Mts, Northern slopes between Mt Dombe & Mt Peza, 19.744972°S, 33.015722°E, 1473 m, 17 April 2014, *A. Mapaura 658* (SRGH!); Manica District, Chimanimani Mts, Small hill below Mt Nhamudimu, 19.755750°S, 33.092667°E, 1722 m, 21 April 2014, *J. Timberlake 5970* (BR!, K!, LMA, SRGH); Báruè District, Serra Choa, 21 km from Catandica (Vila Gouveia), 1400 m, 28 March 1966, *Torre & Correia 15439* (BR, LD, LISC, LMA, MO, UPS, WAG). **Sofala Provinve:** Mt Gorongosa, Gogogo Summit Area, c. 1780 m, January 1972, *K.L. Tinley 2287* (K!, SRGH); Mt Gorongosa, Plateau above western slope Nhamassa Valley, 18.425700°S, 34.047133°E, 1690 m, 19 April 2007, *P. Ballings & B.T. Wursten 550* (LMU!)

South Africa, **KwaZulu-Natal:** [without stated locality but probably Noodsberg (Burtt, 1988; Browning & Gordon-Gray, 1996)], *J. Buchanan 354* (K000244892!); Lions River District, Karkloof, 1525 m, 18 February 1967, *E.J. Moll 3481* (K!, PRE); Ngotshe District, Ngome, 1070 m, 1 April 1977, *O.M. Hilliard & B.L. Burtt 9927* (GENT!, K!); New Hanover District, Little Noodsberg, 24 April 1981, *O.M. Hilliard & B.L. Burtt 14504* (K!); New Hanover District, Little Noodsberg, 1065 m, 12 February 1982, *O.M. Hilliard & B.L. Burtt 15468* (GENT!); Umzinyathi District, Dundee, Mountain View, 11 March 1993, *J. Browning 531* (GENT!, NU). **Limpopo:** Tzaneen District, Wolkberg, 1650 m, 24 April 1971, *P.J. Muller & J.C. Scheepers 185* (K!, PRE); Tzaneen District, Wolkberg, 1615 m, 12 March 1981, *O.M. Hilliard & B.L. Burtt 14315* (K!); Tzaneen District, Lekgalameetse Nature Reserve, 1680 m, 21 January 1986, *M. Stalmans 1007* (K!, PRE). **Mpumalanga:** Barberton District, Saddleback [Hill], 1370 m, 1891, *E.E. Galpin 1316* (K!); Barberton District, 915-1220 m, June 1924, *G. Thorncroft 19183* (K!); Barberton District, 915–1220 m, June 1924, *G. Thorncroft 19184* (K!); Barberton District, Roses Creek, January 1925, *G. Thorncroft 19* (K!); Barberton District, SW of Agnes [Gold] Mine, 08 March 1956, *R. Story 5447* (K!, PRE); Lydenburg District, Buffelskloof Nature Reserve, *C. Reid & J.E. Burrows 1739* (GENT!, K!, PRE); Pilgrim’s Rest District, Mt Anderson, 06 February 1961, *R.G. Strey 3534* (K!, PRE); Pilgrim’s Rest District, Mt Anderson, 16 February 1992, *C. Reid 1754* (GENT!, PRE); Sabie District, Mac Mac Pools, March 1973, *T.H. Arnold 337* (K!, PRE); Sabie District, Witklip summit, 08 March 1981, *O.M. Hilliard & B.L. Burtt 14279* (K!); Sabie District, Long Tom Pass, 1900–2000 m, 02 February 1959, *E. Werdermann & H.-D. Oberdieck 2137* (B, K!); Sabie District, Long Tom Pass, 15 March 1981, *O.M. Hilliard & B.L. Burtt 14362* (K!).

Swaziland, **Hhohho Region:** Mbabane, 1370 m, 12 February 1958, *R.H. Compton 27527* (K!, PRE); Piggs peak, 1220 m, 21 February 1962, *R.H. Compton 31314* (K!, PRE).

ZIMBABWE, **Manicaland:** Chimanimani District, Chimanimani Mts, 1680 m, 19 August 1954, *H. Wild 4565* (K!, SRGH); Chimanimani District, Chimanimani Mts, 1680 m, 09 April 1958, *I.A. Whellan 1526* (K!, SRGH); Chimanimani District, Glencoe Forest Reserve, April 1955, *F.B. Armitage 77/55* (K!, SRGH); Chimanimani District, Mt Pene, 1725 m, 14 April 1957, *N.C. Chase 6403* (K!, SRGH); Chimanimani District, Mt Pene, 1830 m, 06 May 1958, *N.C. Chase 6884* (K!); Chimanimani District, Chimanimani Mts, 2000 m, 30 December 1964, *E.A. Robinson 6333* (K!); Chimanimani District, 02 June 1966, *J.P. Loveridge 1623* (K!, SRGH); Chimanimani Mts, 1850 m, 06 November 1972, *B.K. Simon 2277* (K!, SRGH); Chimanimani District, Chimanimani National Park, Terry’s Cave (19°48′51″S 32°02′00″E), *J. Browning 579* (NU); Mutare District, Banti North, 2130 m, 05 March 1954, *H. Wild 4520* (K!, SRGH); Mutare District, Vumba Mts, Castle Beacon, 1860 m, 04 February 1990, *J.* *Browning 296* (GENT!, NU); Mutare District, Vumba Mts, Castle Beacon (19°06′50″S 32°44′30″E), *B.S. Fisher 1643* (NU).

**12a. *Costularia pantopoda*** (Baker) C.B.Clarke ex Cherm. var. ***pantopoda***

Madagascar, **Antananarivo:** Vakinankaratra, Mt Ibity, 2000 m, February 1914, *H. Perrier de la Bâthie 2740* (MNHN-P-P01936526!); Ambohimandroso, 1500 m, December 1955, *J.M. Bosser 8867* (MNHN-P-P01898166!). **Fianarantsoa:** Ihorombe, Ivohibe RS, 1500–2000 m, 05–11 November 1924, *H. Humbert 3349* (MNHN-P-P01898159!, MNHN-P-P01898158!); Haute Matsiatra, Andringitra Mts, 2400 m, February 1922, *H. Perrier de la Bâthie 14565* (MNHN-P-P01898146!, MNHN-P-P01898147!, MNHN-P-P01898148!, TAN); Haute Matsiatra, Andringitra National Park, Pic Boby, 2500 m, 12 March 1970, *J.-L. Guillaumet 3562* (MNHN-P-P01936527); Haute Matsiatra, Andringitra National Park, 22°08'12.5"S, 46°51'59.9"E, 2190 m, 16 September 2006, *F. Almeda et al. 9368* (K!); Andringitra National, circuit Pic Boby, Andoharina, 22°10'03"S, 46°53'49"E, 2078 m, 24 November 2009, *F. Rakotonasolo et al. 1499* (K000664085!, TAN); Andringitra National Park, Diavolana Trail, last stairway to top of mountain, 22°07'22.9"S, 46°52'08.9"E, 2140 m, 18 April 2010, *I Larridon et al. 2010-0144* (BOL!, GENT!, K!, UPOS!, TAN!).

**13. *Costularia purpurea*** Cherm.

Madagascar, **Antananarivo:** Analamanga, Manankazo, N of Ankazobe, November 1913, *H. Perrier de la Bâthie* *2705* (MNHN-P-P00459993!, MNHN-P-P00459994!); Analamanga, Anjozorobe, near Analabe, December 1958, *J.M. Bosser 12396* (MNHN-P-P01898122!, MNHN-P-P01898123!); Analamanga, Réserve Spécial Ambohitantely, 18°10'42"S, 47°17'00"E, 1530 m, 23–24 November 1993, *G.E. Schatz et al. 3567* (EIU, K!, MO, P, TAN); Mandraka, October 1901, *A.C. D'Alleizette 462* (MNHN-P-P01898170!). **Antsiranana:** Masoala, 500 m, October 1912, *H. Perrier de la Bâthie 2574* (MNHN-P-P00459981!, MNHN-P-P00459982!); Sava, Andapa, Foret Domaniale de Masiaposa, 14°39'20"S, 49°42'20"E, 890 m, 10 November 1995, *D. Ravelonarivo & P. Lowry 885* (K!, MO); Marojejy, 1500–1700 m, 15–25 December 1948, *H. Humbert 22604* (MNHN-P-P01868304!); Sava, Andapa, Doany, Betsomanga, Marojejy, 14°26'S, 49°37'E, 1200 m, 25 October 2001, *L. Gautier et al. 3934* (MNHN-P-P01866862!); Sava, Andapa, Doany, Andranomololo, 14°23'02"S, 49°21'46"E, 1240 m, 25 April 2006 – 8 May 2006, *C. Rakotovao 3033* (BR0000013180720!, G00309607!, MO, MNHN-P-P01868303!, TAN); Sava, Vohemar, Daraina, Binara forest, 13°14'01"S, 49°35'35"E, 1160 m, 9 December 2005, *L. Nusbaumer & P. Ranirison 1754* (G!, K!, MNHN-P-P01866844!, MNHN-P-P01866846!); Sava, Vohemar, Daraina, 13°16'34"S, 49°36'12"E, 920 m, 27 November 2005, *L. Nusbaumer & P. Ranirison 1677* (G!); Sava, Makirovana, NE of Sambava, 14°10'00"S, 49°56'52"E, 805 m, 7 May 2010, *R.H. Archer 3836* (K!, MO, PRE); Diana, Sorata, 13°43'53"S 049°23'41"E, 1594 m, 27 October 2007, *O. Randriambololomamonjy 143* (MNHN-P-P01786001!, TAN); Ambahatra, 13°59'00"S, 48°26'00"E, 1590 m, 15 March 1999, *L. Gautier et al. 3577* (K!, MNHN-P-P01898162!); Bekolosy, 14°02'00"S, 48°18'00"E, 1150 m, 19 May 1995, *L. Gautier & C. Chatelain 2706* (K!, WAG0024581!); Antsatrotro, 14°05'00"S, 48°23'00"E, 1300 m, 24 May 1995, *L. Gautier & C. Chatelain 2774* (G, K!, MNHN-P-P01868300!, TAN). **Fianarantsoa:** Amoron'i Mania, Ambohimitombe forest (Tanala), 1350–1440 m, 23 November 1894, *C.I. Forsyth Major 207* (K!); Amoron'i Mania, Ambohimitombe forest (Tanala), Ambohimitombe forest (Tanala), 1350–1440 m, 23 December 1894, *C.I. Forsyth Major 239* (K!); Kalambatitra AP, Mt Analatsitendrika, 1750 m, November 1933, *H. Humbert 11968bis* (MNHN-P-P01898118!); Kalambatitra AP, Mt Analatsitendrika, 1650–1850 m, November 1933, *H. Humbert 11912bis* (MNHN-P-P01898126!); Kalambatitra AP, Manambolo valley, tributary of the Ionaivo, 1200–1400 m, November 1933, *H. Humbert 12102* (MNHN-P-P01898142!); Andrambovato, 800–1000 m, 24–25 January 1955, *H. Humbert & R.P.R. Capuron 28485* (MNHN-P-P01868298!); Ivohibe AP, 1200 m, November 1924, *W. Armand 74* (MNHN-P-P01898127!, MNHN-P-P01898128!, MNHN-P-P01898141!); Ivakoany, 1250–1550 m, November–December 1933, *H. Humbert 12208* (MNHN-P-P01898130!); Vatovavy-Fitovinany, Ranomafana NP, Sahamalaotra Circuit, 21°14'09.1"S, 47°23'46.9"E, 1125 m, 21 April 2010, *Larridon et al. 2010-0219* (GENT!, TAN); Itomampy, Mt Papanga, near Befotaka, 1300–1700 m, 2–3 December 1928, *H. Humbert 6924* (K!, MNHN-P-P01898119!, MNHN-P-P01898120!, MNHN-P-P01898121!, TAN). **Toamasina:** Lac Alaotra, ANK 55 (MNHN-P-P01898174!); Andrangovalo, SE of Lake Alaotra, 1500–1580 m, October 1937, *H. Humbert & G. Cours 17818* (MNHN-P-P01868301!, MNHN-P-P01868302!); Ankaraoka, SE of Lake Alaotra, 1200–1400 m, October 1937, *H. Humbert & G. Cours 17471* (MNHN-P-P01898125!); Analanjirofo, Ambatoledama, 15°17'S, 50°00'E, June 2003, *P. Antilahimena 2010* (MO, TAN!); Alaotra-Mangoro, Ambohilero, 17°59'21"S, 48°36'34"E, 1165 m, 27 September 2005, *P. Lowry et al. 6581* (MNHN-P-P06242322!); Alaotra-Mangoro, Ambatovy, 18°49'58"S, 48°19'46"E, 1061 m, 11 March 2005, *C. Rakotovao 1619* (TAN!); Alaotra-Mangoro, E of Tana on road to Perinet, forest E of Moramanga, 18°58'S, 48°22'E, 1000 m, 3 April 1987, *P. Phillipson 1628* (K!, MO, P). **Toliara:** Fort-Dauphin, Ranopiso, 200–1000 m, 25 September 1928, *H. Humbert 5883* (K!, K000244889!, MNHN-P-P01868306!, MNHN-P-P01868307!, TAN, US); Beampingaratra, 800–1500 m, 31 October 1928-1 November 1928, *H. Humbert 6332bis* (MNHN-P-P01868305!). **Location unclear:** East Imerina, Andriangalaotra, November 1880, *J.M. Hildebrandt 3752a* (K!).

**14. *Costularia robusta*** (Cherm.) Larridon

Madagascar, **Antsiranana:** Diana, [Tsaratanana Reserve, Maromokotro], April 1924, *H. Perrier de la Bâthie 16095bis* (MNHN-P-P01868035!); Diana, [Tsaratanana Reserve, Maromokotro], April 1924, *H. Perrier de la Bâthie 16090A* (MNHN-P-P01868286!); Diana, [Tsaratanana Reserve, Maromokotro], April 1924, *H. Perrier de la Bâthie 16398* (P, not seen); Diana, [Tsaratanana Reserve, Maromokotro], 2600–2800 m, November–December 1937, *H. Humbert 18385* (MNHN-P-P01868270!); Diana, [Tsaratanana Reserve, Maromokotro], 2600–2800 m, November–December 1937, *H. Humbert 18386* (MNHN-P-P01898138!); Tsaratanana AP, 1400–1800 m, November–December 1937, *H. Humbert 18214* (MNHN-P-P01898156!, MNHN-P-P01898164!); Tsaratanana AP, 2300 m, November–December 1937, *H.* *Humbert 18491* (MNHN-P-P01898165!); Diana, [Tsaratanana Reserve, Maromokotro], 14°04'19"S, 48°58'58"E, 2865 m, 21 April 2003, *L.J. Razafitsalama et al. 431* (MO, MNHN-P-P01868033!, TAN); [locality description unclear but close to Tsaratanana Reserve], 14°12'00"S, 49°06'30"E, 2000–2301 m, *Callmander et al. 445* (MO, MNHN-P-P01898079!); Ambohimirahavavy, Montagnes au Nord de Mangindrano, 1951, *H. Humbert 25320* (MNHN-P-P01866847!); Partie occidentale du massif de Marojejy (Nord-Est) de la vallée de l'Ambatoharanana au bassin supérieur de l'Antsahaberoka, 1400 m, 9 November – 2 December 1959, *H. Humbert 31646* (MNHN-P-P01898140!); Partie occidentale du massif de Marojejy (Nord-Est) de la vallée de l'Ambatoharanana au bassin supérieur de l'Antsahaberoka, 1600–1800 m, 15–25 November 1959, *H. Humbert 31821* (MNHN-P-P01898139!); Marojejy, 2000–2137 m, November 1973, *P. Morat* *4103* (MNHN-P-P01898168!); Vallée inférieure de l'Androranga, affluent de la Bemarivo (Nord-Est) aux environs d'Antongodriha, Massif de Betsomanga, 1300–1350 m, 17–20 November 1950, *H. Humbert & R. Capuron 24345* (MNHN-P-P01898136!).

**15. *Costularia xipholepis*** (Baker) Henriette & Senterre

Seychelles, **Mahé:** Copolia, 500 m, 4.64916°S, 55.45778°E, 17 July 2014, *B. Senterre & E. Henriette 7101* (GENT!, P!, SEY!), 25 August 2006, *M. Luceño & M. Guzmán 8406 ML* (UPOS!); Pérard, pentes nord-ouest, 821 m, 4.64011° S, 55.43617° E, 17 March 2013, *B. Senterre & E. Henriette 6558* (P!, SEY!), 19 May 2013, *B. Senterre 6583* (P!, SEY!), 18 June 2013, *B. Senterre 6586* (GENT!, P!, SEY!), 26 July 2013, *B. Senterre 6589* (GENT!, P!, SEY!), 29 January 2014, *B. Senterre & E. Henriette 6964* (GENT!, P!, SEY!), 11 December 2014, *B. Senterre & E. Henriette 7117* (SEY!).
