# Supplementary figures and images for: Revision of the Afro-Madagascan genus Costularia (Schoeneae, Cyperaceae): infrageneric relationships and species delimitation
Source: PeerJ. 2019 Feb 27;7:e6528. doi: 10.7717/peerj.6528 (PMC6397637; doi:10.7717/peerj.6528)

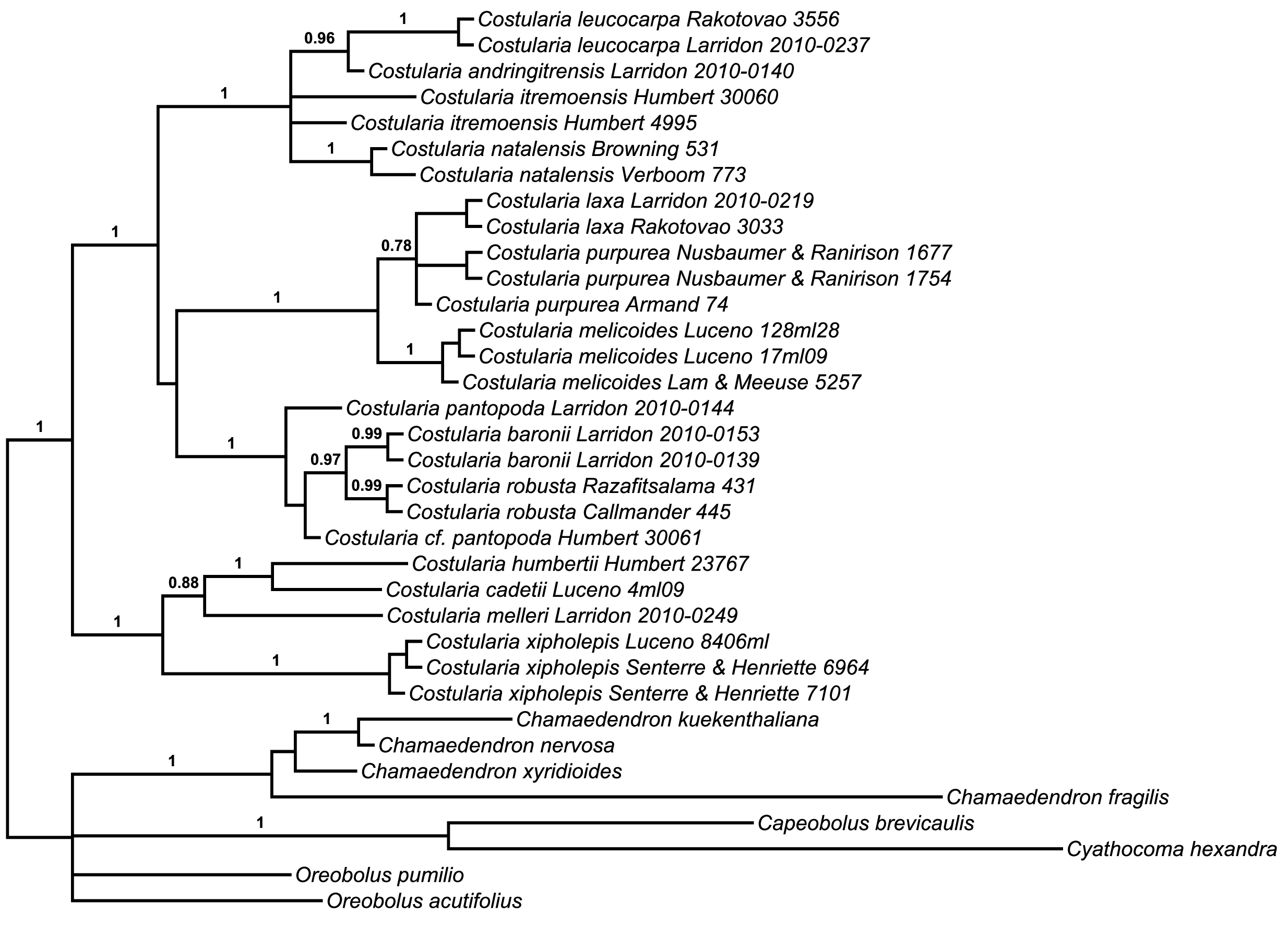

Supplement: Supplemental Information 4 — Only posterior probabilities above 0.7 are shown. [file peerj-07-6528-s004.png]

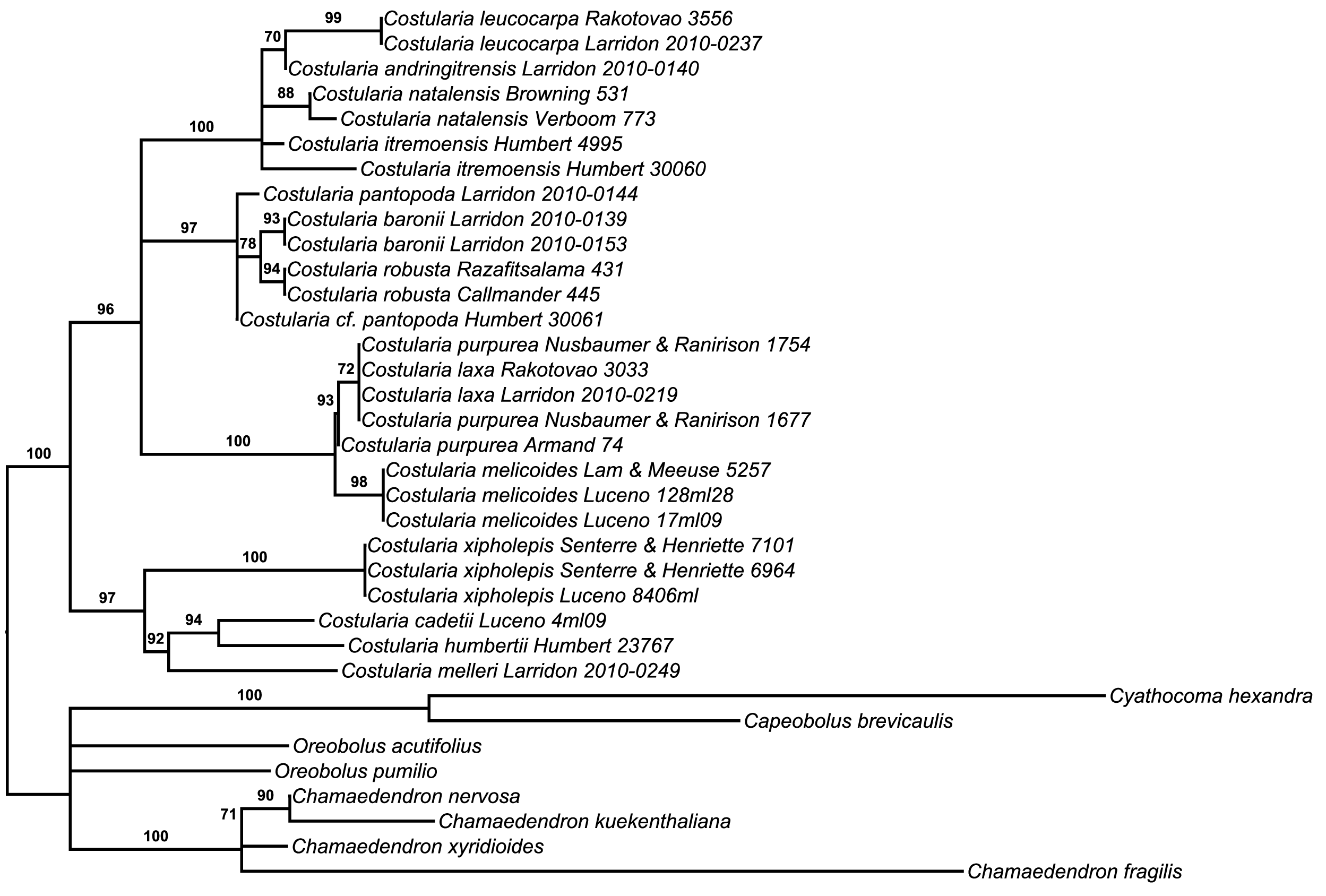

Supplement: Supplemental Information 5 — Only bootstrap values above 70% are shown. [file peerj-07-6528-s005.png]

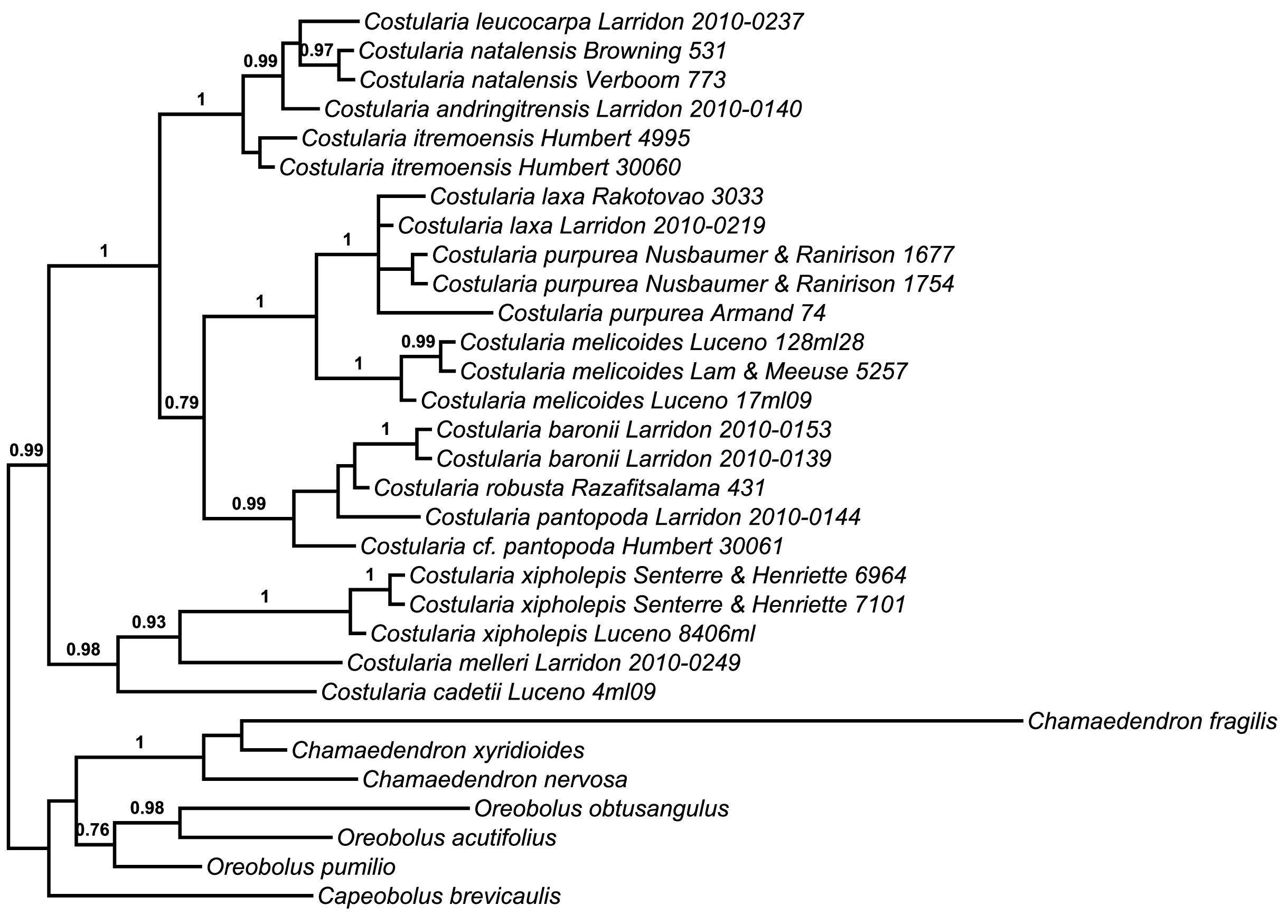

Supplement: Supplemental Information 6 — Only posterior probabilities above 0.7 are shown. [file peerj-07-6528-s006.png]

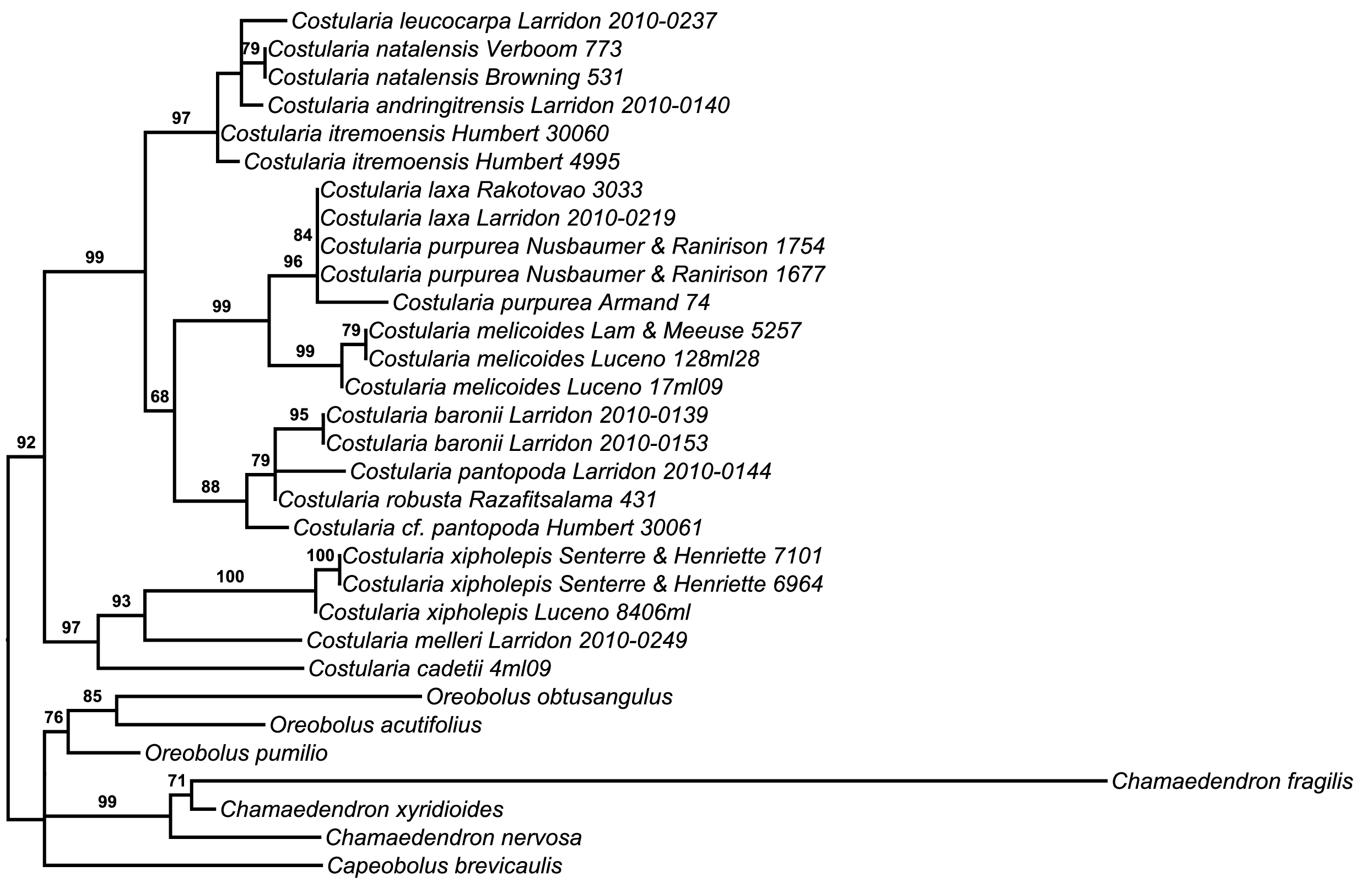

Supplement: Supplemental Information 7 — Only bootstrap values above 70% are shown. [file peerj-07-6528-s007.png]

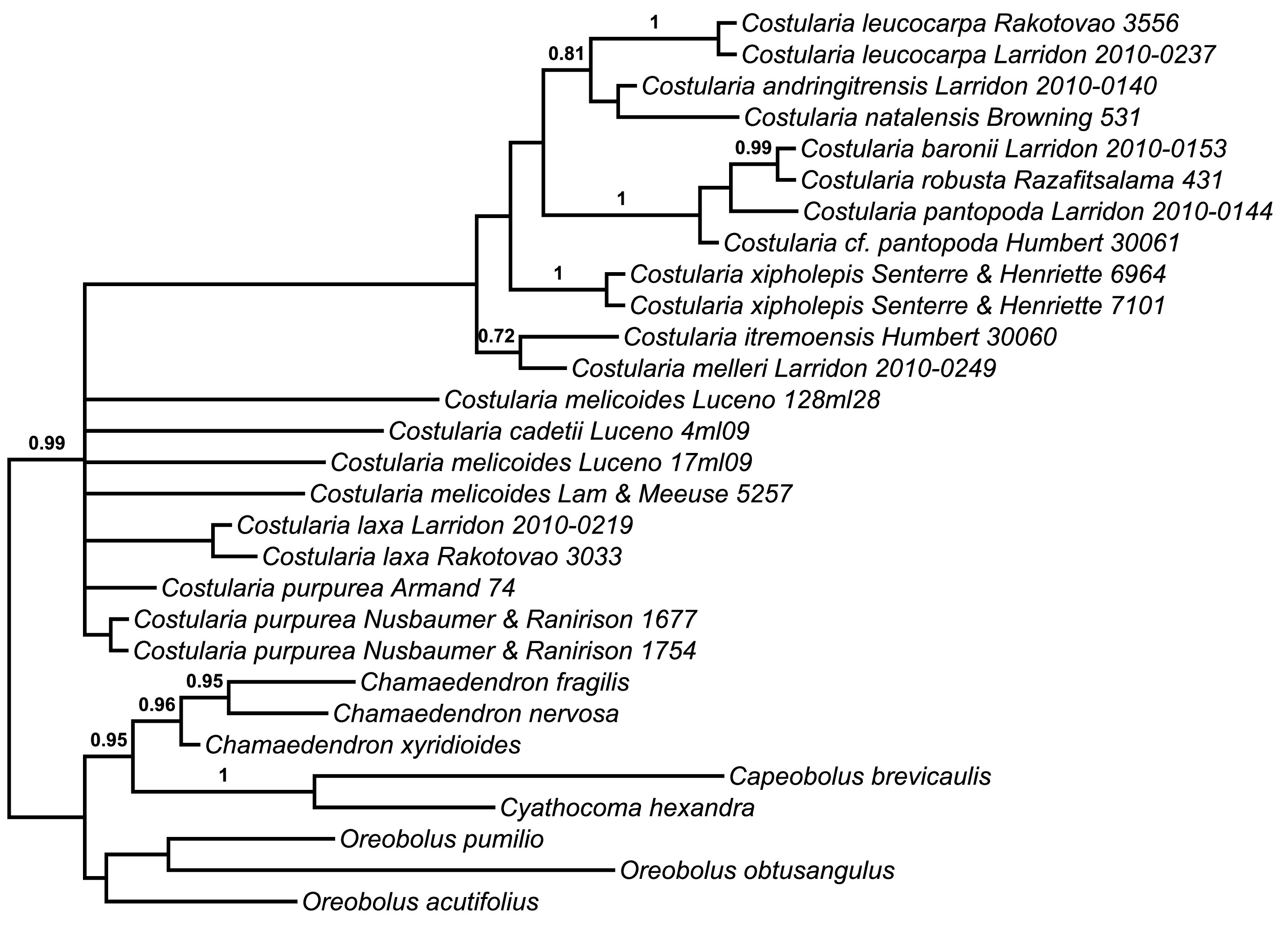

Supplement: Supplemental Information 8 — Only posterior probabilities above 0.7 are shown. [file peerj-07-6528-s008.png]

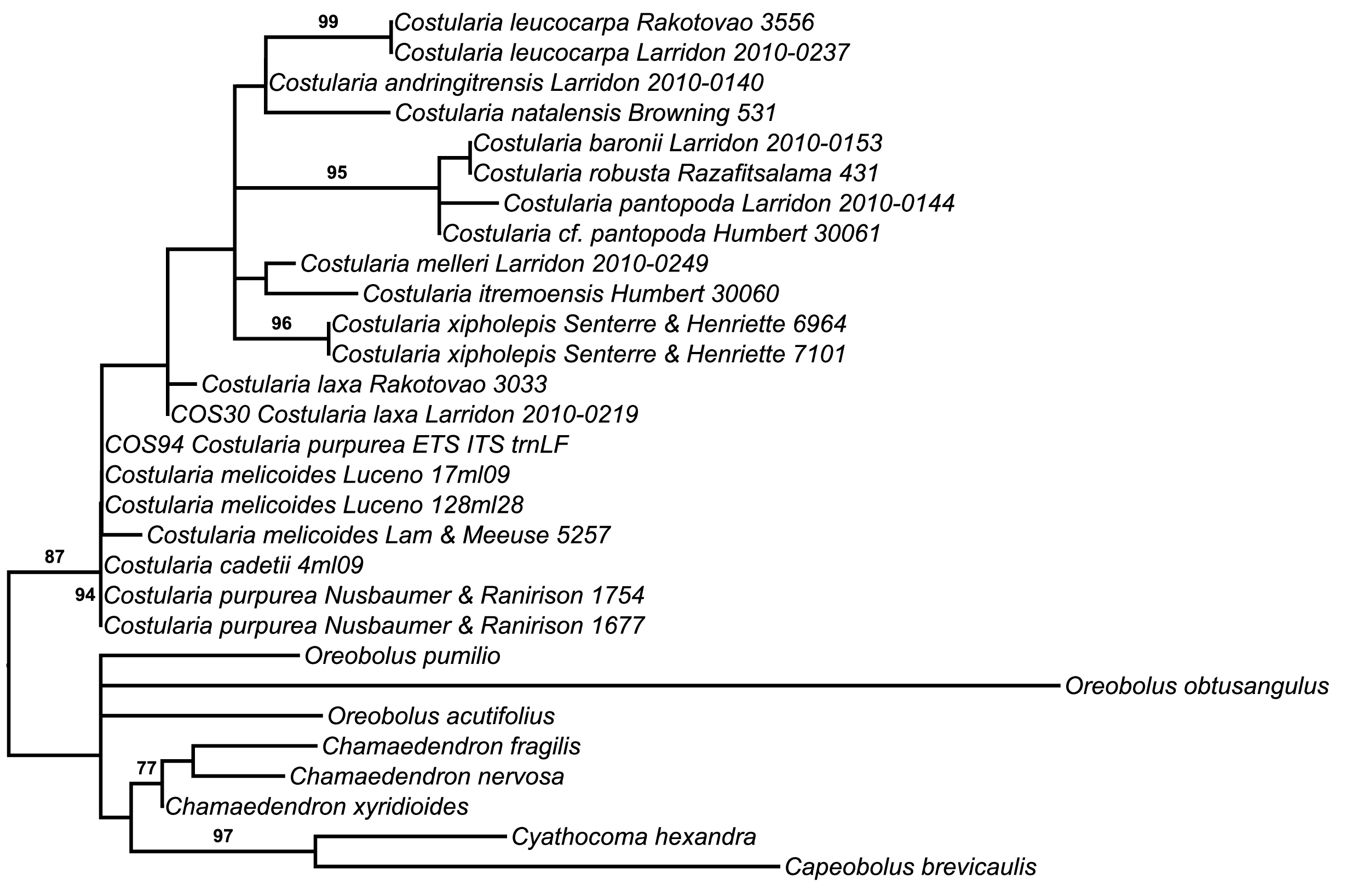

Supplement: Supplemental Information 9 — Only bootstrap values above 70% are shown. [file peerj-07-6528-s009.png]

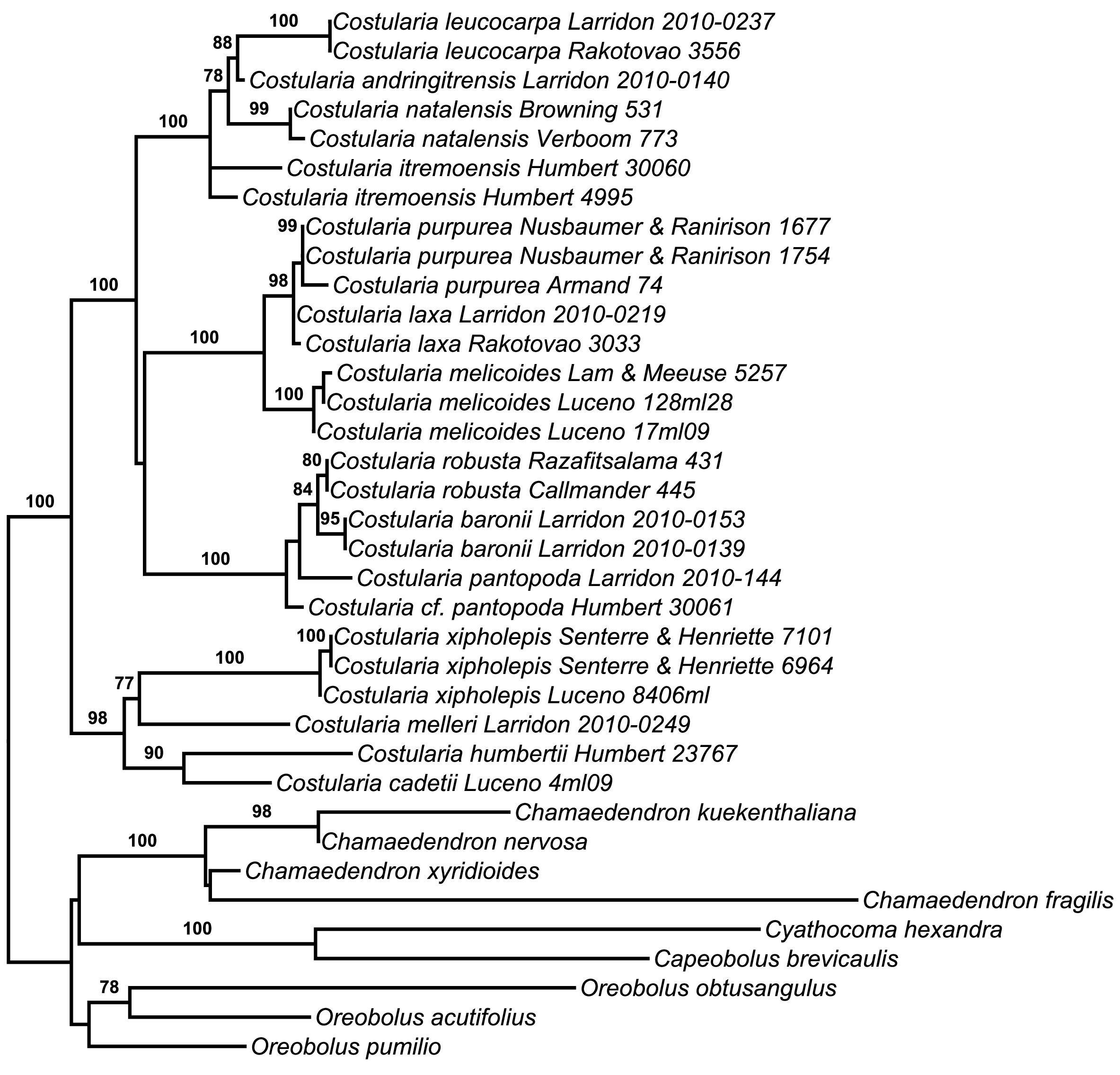

Supplement: Supplemental Information 10 — Only bootstrap values above 70% are shown. [file peerj-07-6528-s010.png]
